# Design of High Performance Scroll Microcoils for Nuclear Magnetic Resonance Spectroscopy of Nanoliter and Subnanoliter Samples

**DOI:** 10.3390/s21010170

**Published:** 2020-12-29

**Authors:** Meriam Khelifa, Denis Mounier, Nourdin Yaakoubi

**Affiliations:** 1 Environnement Méditerranéen et Modélisation des Agro-Hydrosystèmes (EMMAH) UMR 1114, Université d’Avignon et des Pays de Vaucluse, 84018 Avignon, France; 2 Laboratoire d’Acoustique de l’Université du Mans (LAUM) UMR 6613, Le Mans Université, Avenue Olivier Messiaen, 72085 Le Mans, France; Nourdin.Yaakoubi@univ-lemans.fr; 3 Institut des Molécules et Matériaux du Mans (IMMM) UMR 6283, Le Mans Université, Avenue Olivier Messiaen, 72085 Le Mans, France; denis.mounier@univ-lemans.fr

**Keywords:** nuclear magnetic resonance spectroscopy, finite element modelling, design of experiment, scroll coil, optimisation

## Abstract

The electromagnetic properties of scroll microcoils are investigated with finite element modelling (FEM) and the design of experiment (DOE) approach. The design of scroll microcoils was optimized for nuclear magnetic resonance (NMR) spectroscopy of nanoliter and subnanoliter sample volumes. The unusual proximity effect favours optimised scroll microcoils with a large number of turns rolled up in close proximity. Scroll microcoils have many advantages over microsolenoids: such as ease of fabrication and better $B_{1}$-homogeneity for comparable intrinsic signal-to-noise ratio (SNR). Scroll coils are suitable for broadband multinuclei NMR spectroscopy of subnanoliter sample.

## 1. Introduction

Nuclear Magnetic Resonance (NMR) spectroscopy is a widely used analytical technique of substances despite its low sensitivity compared to other methods such as mass spectrometry or optical spectrometry. One key to enhancing mass sensitivity is to use microcoils and thus small sample volumes [1,2,3,4,5]. For microsolenoids with a diameter greater than 100 μm, the limit of detection (LOD) of mass-limited samples is inversely proportional to the coil diameter [6,7,8,9]. Using spiral planar microcoils with an inner diameter of 50 μm and a 15pL sample (a very small fish egg), the mass sensitivity is multiplied by a factor of 3000 compared to a conventional 5 mm NMR probe [10]. Microsolenoids are suitable for magnetic resonance imaging (MRI) of small samples with picoliter resolution [3,11]. The miniaturization of coil and sample not only improves sensitivity, but opens the door to many applications of NMR spectroscopy with low-cost portable NMR spectrometers [9,12].

**Figure 1:**
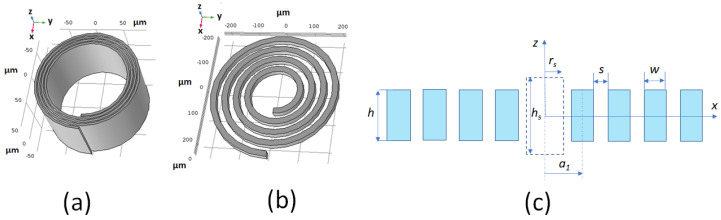
(**a**) Scroll microcoil. (**b**) Spiral planar microcoil. (**c**) Coil cross-section showing the geometrical parameters of the coil and the cylindrical sample centred in the coil (dashed lines).

Microsolenoids are the most widely used microcoils in both commercial [13] and laboratory made NMR systems. The highest performance is achieved when the size of the receiving coil is perfectly matched to the sample volume [14]. To enhance the filling factor, other coil designs can be used, such as striplines [14,15]. Planar spiral microcoils (Figure 1b) have a poor filling factor, but are suitable for NMR broadband spectroscopy [16,17] and NMR imaging [18]. Recently, CMOS-based NMR probes emerged, where planar microcoils are directly fabricated on the CMOS-chip [19,20]. Unlike conventional NMR systems, CMOS-NMR probes are designed to work within the static magnetic field ($B_{0}$-field) and allow the acquisition of NMR spectra of nanoliter and subnanoliter samples [21,22].

Scroll coils were proposed for NMR spectroscopy by Grant et al. [23]. This type of coils, which are shown in Figure 1a, were proved to have a better performance than solenoids at high frequencies [24]. Similar to the spiral planar coil, the geometry of a scroll coil is defined by the parameters which are shown in Figure 1c. The trace height *h* of a scroll coil is much greater than the trace width *w* and the spacing *s* between conductors may be small compared to *w*. The cylindrical geometry of a scroll coil favours a good homogeneity of the magnetic field inside the coil (the $B_{1}$-homogeneity). Scroll coils could be fabricated from a thin bilayer of conductor/isolator rolled up into a cylindrical spiral. Scroll microcoils could be integrated to the electronic chips (MEMS) in a manner similar to micro-solenoids [11]. Batch fabrication of scroll microcoils would be possible, thus allowing for the use of multicoil array for parallel spectrum acquisitions [25,26,27,28]. Also, scroll microcoils could be integrated in a micro-total analysis system (μTAS) or in a microfluidic system [29].

Optimising coil performance for NMR spectroscopy is a complex problem because of the numerous parameters to optimise simultaneously [16]: (i) signal-to-noise ratio (SNR), (ii) $B_{0}$-homogeneity, (iii) $B_{1}$-homogeneity, (iv) coil resistance *R*, (v) self-resonance frequency. Maximizing SNR means minimising the time of measurement and the LOD of a substance [30].

Maximising “$B_{0}$-homogeneity” is mandatory to obtain high resolution NMR spectra. Maximising “$B_{1}$-homogeneity” is necessary to maximise SNR and to obtain high resolution spectra. The resistance is generally not a parameter to optimize when the coil is connected to the low noise amplifier (LNA) through an impedance transformer which matches the coil impedance to a 50Ω cable and tunes the NMR probe at the Larmor frequency of the nucleus. But in broadband multinuclei NMR, the coil can be directly connected to the low noise amplifier without impedance matching and without tuning. So it is important that the coil resistance is not too low in order to preserve the intrinsic SNR of the coil at the output of the LNA [16,20]. Rejecting “self-resonance frequency” at the GHz range allows for broadband multi-nuclei spectroscopy [16,17]. Fortunately, miniaturization is beneficial to several criteria simultaneously. SNR increases as the dimensions of coil and sample decrease. Also, it is easier to design a magnet with a high $B_{0}$-homogeneity for a small sample volume [12].

We present an approach to optimize the design of scroll microcoils for NMR spectroscopy of nanoliter and subnanoliter sample volume. The method combines finite element modelling (FEM) and design of experiment (DOE). We present in Section 2 the criteria of coil performance and the method used to calculate them. In Section 3, we present the method of optimisation using the design of experiment approach. We illustrate the method for a sample of 1 nL at 200 MHz. We compare the performance of a scroll microcoil with a solenoid of similar dimensions. Finally, in Section 4, we discuss the unusual proximity effect on AC resistance of scroll coils.

## 2. Methods

### 2.1. Responses of Interest for Coil Performance

#### 2.1.1. Signal-to-Noise Ratio

**Figure 2:**
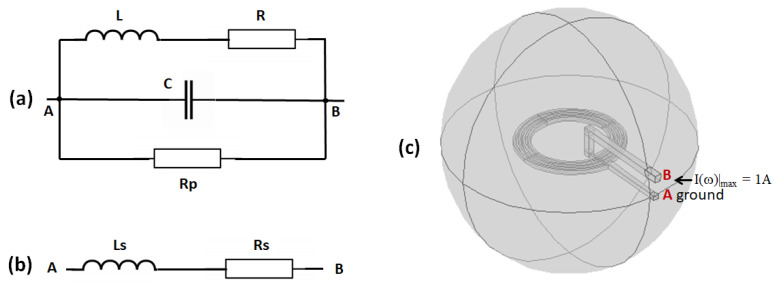
(**a**) Electrical model of a coil. (**b**) Equivalent serial model. (**c**) Finite element model (FEM) of a microcoil.

The coil is the first stage of the measurement chain in a NMR system. So, it is of primary importance to design a microcoil with the highest signal-to-noise ratio (SNR) [20,31]. Subsequent stages of the measurement chain can only deteriorate the intrinsic SNR of the coil. The electrical model of the coil is shown in Figure 2a. The resistance *R* takes only into consideration the losses within the conductor. The self-capacitance *C* and the self-inductance *L* of the coil determine the self-resonance frequency $f_{r}=1/\left(2\pi \right)/\sqrt{LC}$. A resistance $R_{p}$ in parallel with *C* takes account of possible capacitor losses due to the lossy dielectric filling the gap between the conductors [32]. Due to the large area of the conductive surfaces facing each other in a scroll coil, the self-capacitance of a multi-turns scroll microcoil is of the order of 0.1pF (see Section 2.2.4), which is higher than the self-capacitance of micro-solenoids and spiral planar coils. So the capacitor losses in a scroll microcoil are expected to be larger than in a microsolenoid or a planar microcoil of comparable dimensions. Fortunately, a rapid evaluation of dielectric losses (Section 2.2.4) shows that they are indeed negligible compared to the conductor losses. Also, sample losses are represented by a resistance $R_{sample}$ in series with *R* (not represented in Figure 2a). The resistance $R_{sample}\propto a^{2}$ where *a* is a characteristic dimension of the coil (assuming that the sample totally fills the interior of the coil) [33]. In Reference [33], $R_{sample}\approx 1\Omega$ for a sample of electrical conductivity $1\;S\;m^{-1}$ and a coil with a≈10 mm, so for a microcoil with a≈0.1 mm, the serial resistance $R_{sample}\approx 10^{-4}\Omega$, which is negligible compared to a microcoil resistance. In conclusion, the resistances $R_{p}$ and $R_{sample}$ can be ignored.

At the working frequency f=ω/(2π), well below the self-resonance frequency $f_{r}$ of several GHz, a typical 10-turn scroll microcoil has: R≈1Ω, L≈10nH and C≈0.1pF, so that at f=200 MHz, $R<<L\omega <<1/\left(C\omega \right)<<R_{p}$. In these conditions, the coil behaves as an inductive dipole and the electrical model can be simplified by the serial model which is shown in Figure 2b with the self-inductance $L_{s}\approx L/\left[1-{\left(f/f_{r}\right)}^{2}\right]$ in series with the resistance $R_{s}\approx R/\left[1-{\left(f/f_{r}\right)}^{2}\right]$. The electrical parameters of microcoils can be calculated by using finite element modelling (FEM) with the software COMSOL Multiphysics^®^ (COMSOL Inc., Stockholm, Sweden). Figure 2c shows a 3D-FEM of a planar microcoil, where only the conductor is modelled.

The self-inductance *L* and the serial resistance *R* determines the quality factor Q=Lω/R of the oscillating circuit tuned at the Larmor angular frequency ω. The coil resistance *R* determines the noise level in NMR signals. At temperature *T*, the main noise contribution is Johnson’s noise of the coil. This is a white noise of power density $4k_{B}TR$, where $k_{B}$ is the Boltzmann’s constant. The variance of noise voltage is equal to the spectral power density multiplied by the bandpass Δf of the receiving circuit.

The NMR microcoil is used as an antenna receiving the free induction decay (FID) voltage induced by the precession of nuclei in the static magnetic field $B_{0}$. Prior to recording the FID signal, a radio-frequency (RF) magnetic field $B_{1}$ flips the nuclear magnetization, so that the precession of the nuclear magnetization induces variations of the magnetic flux in the coil.

According to the reciprocity principle [34], the magnetic field $B_{1}$, induced by a current *I* in the coil, determines the amplitude of the FID signal. In a scroll microcoil, $B_{1}$ is almost aligned along the coil axis (*z*-axis), so that the transverse components $B_{x}$ and $B_{y}$ can be neglected. In this context, the $B_{z}$-component is a good approximation of the $B_{1}$ field, so that the SNR is proportional to the following expression [9,29]:

(1) $$\mathrm{SNR}\propto f^{2}\;V_{s}\frac{1}{\sqrt{R}}\;\frac{\langle B_{z}\rangle }{I},$$

where $f=\frac{1}{2\pi }\gamma B_{0}$ is the Larmor frequency and γ is the gyromagnetic ratio of the nucleus, and $\langle B_{z}\rangle$ is the average of $B_{z}$ within the sample.

#### 2.1.2. $B_{1}$- and $B_{0}$-Homogeneity

The homogeneity of the magnetic field $B_{1}$ is an important criterion for coil performance in NMR spectroscopy. The $B_{1}$-homogeneity can be characterized by [35]:

(2) $$H\left(\%\right)=\left(1-\frac{\sigma_{B_{z}}}{\langle B_{z}\rangle }\right)\times 100,$$

where $\sigma_{B_{z}}=\sqrt{\langle {B_{z}}^{2}\rangle -{\langle B_{z}\rangle }^{2}}$ is the standard deviation of $B_{z}$ within the sample. A value H=100% means that $B_{1}$ is perfectly homogeneous, as it would be for a sample inside an infinite solenoid. In practice, H>90% is acceptable. The scroll coil will be optimized for a cylindrical sample of volume $V_{S}=1nL$, of height $h_{s}$ and radius $r_{s}$. To achieve maximum SNR, the inner turn radius of the coil $a_{1}$ must be close to the sample radius $r_{s}$. Moreover, the ratio $h_{s}/r_{s}$ must maximize both SNR and homogeneity *H*. Indeed, it turns out to be impossible to simultaneously maximize SNR and $B_{1}$-homogeneity. To solve the dilemma of SNR and *H* optimisation, we fixed the constraint H>90% for $B_{1}$-homogeneity. Then SNR can be maximized by varying the two parameters $h_{s}/r_{s}$ and $a_{1}$ for a fixed sample volume of 1nL. In order to avoid the overlapping of coil and sample we kept a gap of 10 μm between $a_{1}$ and $r_{s}$. The result of optimisation for the coil inner turn radius is: $a_{1}=70$ μm, and for sample dimensions: $r_{s}=60$ μm, and $h_{s}=88.4$ μm. Concerning the scroll microcoils we simulated, we define the filling factor as the ratio ${\left(r_{s}/a_{1}\right)}^{2}$, which is about 75%. Once the dimensions of the sample and the inner turn radius are fixed, it is possible to optimise the SNR by varying the remaining parameters: *w*, *h*, *s* and the number of turns *N*.

To attain the highest spectral resolution, the $B_{0}$ field must have the highest homogeneity within the sample. In solenoids, the use of a matching fluid is necessary to minimize the deterioration of $B_{0}$-homogeneity close to wires [2]. It is probable that the use of a matching fluid would be required for scroll coils in order to achieve the necessary $B_{0}$-homogeneity. However, the issue of $B_{0}$-homogeneity necessitates simulations that are out of the scope of this paper.

### 2.2. Finite Element Modelling of Coils

Coil modelling was performed using software COMSOL Multiphysics^®^. The AC-DC module of the software is suitable for modelling microcoils at frequencies from 0 to about several GHz, as the vacuum wavelength is much longer than the dimensions of a microcoil. At high frequencies, the skin- and the proximity effects determine the current distribution in the conductor. FEM is capable to accurately evaluate the current distribution, the coil resistance and the magnetic field $B_{1}$ in the sample. The FEM must represent as accurately as possible the physical behaviour of the system coil/sample. Samples with a weak electrical conductivity and a small volume induce negligible losses. This assumption can be verified by simulating a biological sample of 1 nL with an electrical conductivity of $1\;S\;m^{-1}$ [36].

The insulating material separating the conductor in a scroll coil must have low dielectric losses. Polyimid is a good dielectric material, widely used as insulator in electronic circuitry and as substrate in the fabrication of planar microcoils [37]. With a dissipation factor of 0.01 for the insulator, the additional losses due to the insulator are negligible at 200 MHz. In consequence, FEM of microcoils does not need to take into account the insulator but only the conductor.

#### 2.2.1. Current Density

**Figure 3:**
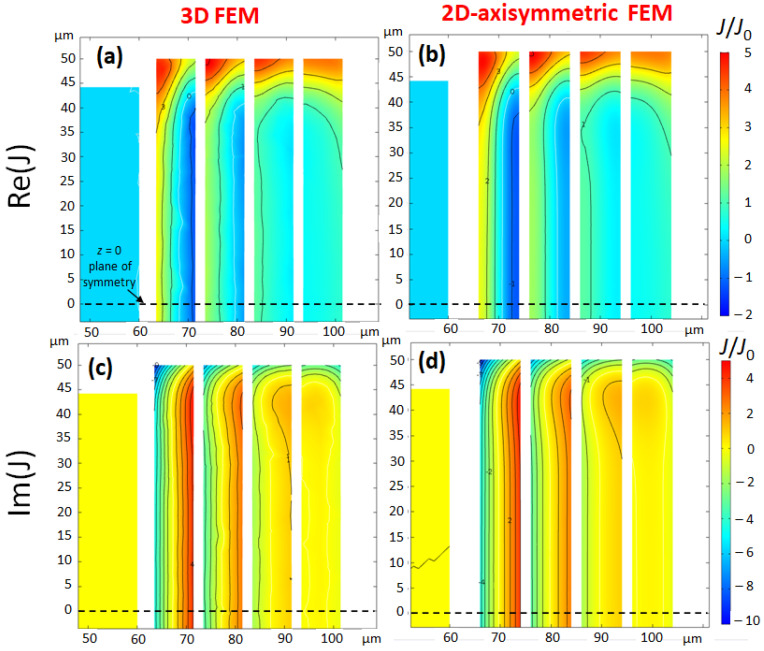
**Coil 1**: Comparison of the complex current density (real part (**a**,**b**)) and imaginary part (**c**,**d**)), for a current excitation: $i\left(t\right)=I_{0}\sin\left(2\pi \;f\;t\right)$, at f=200 MHz, calculated with 3D-FEM (**a**,**c**) and 2D-axisymmetric FEM (**b**,**d**). The geometrical parameters of Coil 1 are: $a_{1}=70$ μm, w=8 μm, h=100 μm, and s=2 μm. The current density is normalized to the average current density $J_{0}=I_{0}/\left(w\;h\right)$. As the plane z=0 is a plane of symmetry of the coil, only the upper half of the coil is drawn. The averages of current densities in a cross-section are respectively $J_{0}$ and zero for the real part and the imaginary part. The contour lines are drawn in steps of one unit of the normalized current density. For the 3D-FEM figures, the cut-plane is the *yz*-plane (the *zx*-plane in Figure 1 contains the input and output ports of the coil).

The distribution of currents within the conductor determines the coil resistance and the magnetic field $B_{1}$. Taking benefit of the almost cylindrical shape of scroll coils, it is advantageous to use 2D-axisymmetric modelling instead of 3D-modelling which requires much more computing resources. However, a 2D-axisymmetric model is an approximation of the real coil, so it is important to verify its accuracy. To compare 2D- and 3D modelling, two test scroll microcoils of 4 turns each, named Coil 1 and Coil 2 were built. For both coils, the parameters are: $a_{1}=70$ μm (inner turn radius), h=100 μm (trace height), and s=2 μm (spacing between conductors), whereas the trace width *w* is 8 μm for Coil 1 and 4 μm for Coil 2. Figure 3 shows the current density of Coil 1 calculated with the 3D- and the 2D-axisymmetric models. Coil 1 is excited by a sinusoidal current $i\left(t\right)=I_{0}\sin\left(2\pi \;f\;t\right)$ of amplitude $I_{0}=1$ A at the working frequency f=200 MHz. The current distribution within the conductor of Coil 1 is very inhomogeneous. This feature occurs when the skin-depth is significantly smaller than the trace width. The current distributions of both models are essentially in agreement, although the contour lines of the 3D-model are less smooth, because of the mesh which is less refined.

The current density has a significant imaginary part superimposed to the real part, which means that the current density is not in phase with the excitation i(t). Another feature of real and imaginary current densities is the presence of a nodal line, i.e., a line where the sign of the current density changes. Also, the current density displays a reinforcement of current at the top and the bottom of the coil.

**Figure 4:**
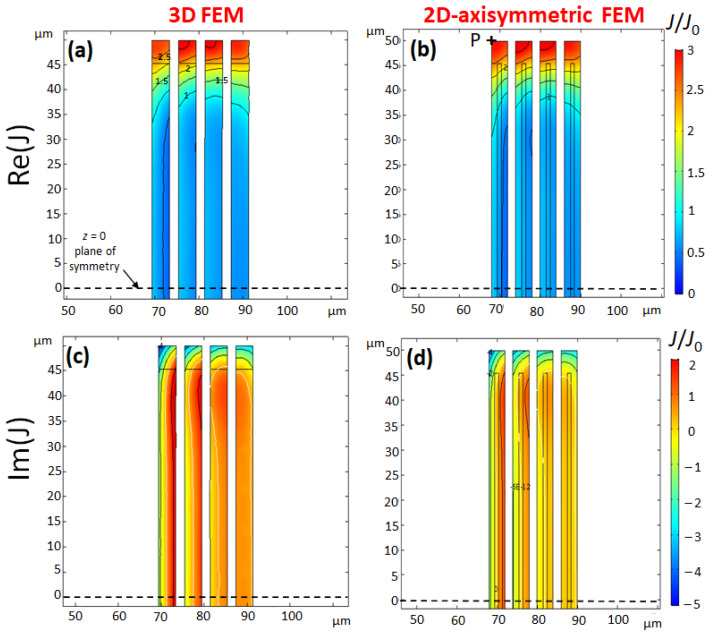
**Coil 2**: Normalised complex current density (real part (**a**,**b**)) and imaginary part (**c**,**d**), for a current excitation: $i\left(t\right)=\;I_{0}\sin\left(2\pi \;f\;t\right)$, at f=200 MHz, calculated with 3D-FEM (**a**,**c**) and 2D-axisymmetric FEM (**b**,**d**). Parameters of Coil 2 are identical to Coil 1 except the trace width w=4 μm. The contour lines of the normalized current density are drawn in steps of 0.5 for the real part (**a**,**b**) and one unit for the imaginary part (**c**,**d**). To interpret the Figures, consider for example the point P of coordinates: (r=68,z=50) μm in Figure 4 (first turn, inner corner), which is a hot spot of current density $J\approx \left(3-5i\right)\;J_{0}$. The modulus is $\left|J\right|\approx 6\;J_{0}$ and $Arg\left(J\right)\approx -60^{\circ }$.

Figure 4 shows the complex current density for Coil 2 at 200 MHz. The predictions of 2D- and 3D-models are in agreement. The reduction by a factor of two of trace width has a drastic effect on the current distribution which is more homogeneous in Coil 2 than in Coil 1. The real part of current density has no more nodal line. Such a behaviour is expected when the thickness *w* of the conductor is around or below the skin-depth δ. For w<<δ, the current density tends to the homogeneity of the DC current. The current density of Coil 2 is reinforced by a factor of 6 at the bottom and the top of the coil compared to average current density $J_{0}$. It is interesting to point out that the distance 100 μm between the top and the bottom of the coil is close to the average coil radius ≈80 μm. The reinforcement of current density has some similarity with the Helmholtz coils configuration. In the middle of the coil, the current density is close to the average current density $J_{0}$. According to the Biot-Savart law and the principle of superposition, the magnetic field of a scroll coil benefits from the hybrid nature of the solenoid and Helmholtz coils.

#### 2.2.2. Coil Resistance

The resistance *R* of the coil is calculated through the time average power $P_{d}$ dissipated in the conductor:

(3) $$P_{d}=\frac{1}{2}\;R\;I_{0}^{2}=\frac{1}{2}\;{\;\int \int \int \;}_{Conductor}\sigma J\;\cdot \;J^{*}\;d\tau ,$$

where σ is the electrical conductivity, and $I_{0}$ is the amplitude of the sinusoidal current. Equation (3) shows that power dissipation in a conductor due to the Joule effect is not sensitive to the phase θ of complex current density $J=\left|J\right|e^{i\theta }$. With COMSOL Multiphysics^®^, the power $P_{d}$ is calculated through an integration of $\sigma J\;\cdot \;J^{*}=\sigma {\left|J\right|}^{2}$ in the volume of the conductor.

The coil is connected to the measurement circuit and the additional resistance due to the connexion wires to the measuring circuit may have drastic negative effects on SNR, especially when the intrinsic coil resistance is very low. At frequencies 0, 100, 200 and 400 MHz, the resistances of a copper wire with a square cross-section 40 μm × 40 μm, of length ≈1 mm are respectively: 13, 30, 43 and 60mΩ. When the skin depth is smaller than half of the wire width, corresponding to frequencies f>20 MHz, the resistance increases proportionally to $1/\sqrt{f}$. An intrinsic coil resistance of at least 100mΩ is required to avoid the negative effect of additional resistance due to the connexion leads, which increases noise and degrades SNR.

#### 2.2.3. $B_{z}$-Magnetic Field

The magnetic field $B_{z}$ determines the amplitude of the NMR signal. According to the Biot-Savart law, the real and imaginary parts of the current density determine respectively the real part and the imaginary part of the magnetic field. Since the average of the imaginary part ℑm(J) is zero in a cross-section, $ℑm\left(B_{z}\right)<<ℜe\left(B_{z}\right)$. The relevant magnetic field to evaluate the amplitude of the NMR signals by Equation (1) is $ℜe(B_{z})$. According to the principle of reciprocity [38], the electromotive force (emf) at the coil terminals is proportional to $B_{z}$. Thus, the main component of the emf is proportional to $ℜe(B_{z})$, whereas $ℑm(B_{z})$ determines the component of the emf which is in quadrature with the main in-phase emf component. In the following, we neglect the weak component $ℑm(B_{z})$ and take into account only $ℜe(B_{z})$ for the evaluation of the SNR.

**Figure 5:**
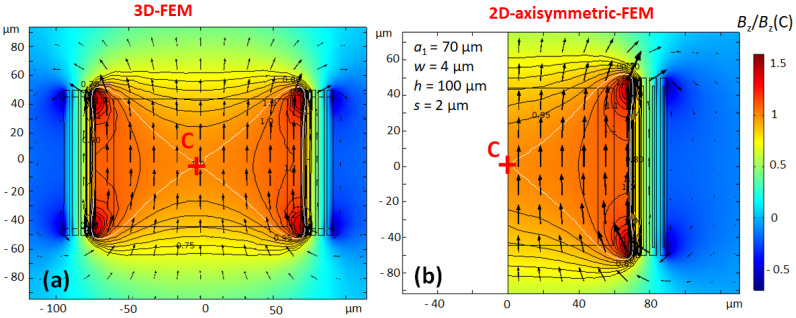
Magnetic field of Coil 2 for (**a**) the 3D-model and for (**b**) the 2D-axisymmetric model. The relative magnitude of $B_{z}$ is normalized to the value $B_{z}\left(C\right)$ at the centre of the coil (point C). The contour plot shows isovalue lines of $B_{z}/B_{z}\left(C\right)$ by steps of 0.05. The sample of 1nL is represented by a rectangle inside the coil. The $B_{1}$-homogeneity can be apreciated by the arrows representing $B_{1}$-vectors.

Figure 5 shows the real part of the magnetic field $ℜe(B_{z})$, which is calculated with 3D- and 2D-axisymmetric models. The predominant direction of the magnetic field in the coil is along the coil axis (*z*-axis). The magnetic field of a cylindrical scroll coil is homogeneous within a 1nL sample.

#### 2.2.4. Coil Self-Inductance, Self-Capacitance and Dielectric Losses

The self-inductance *L* is calculated through the total magnetic energy:

(4) $$E_{m}=\frac{1}{2}L{I_{0}}^{2}$$

The magnetic energy $E_{mi}$ inside the coil is homogeneously distributed and an estimate is: $E_{mi}\approx \left(1/2\right){B_{z}\left(C\right)}^{2}/\mu_{0}\times V_{S}$, where $B_{z}\left(C\right)$ is the magnetic field at the centre of the coil and $V_{S}$ the sample volume, assuming a filling factor of 100%. As the magnetic energy $E_{mi}$ inside a scroll coil is roughly half of the total magnetic energy, a rapid estimate of *L* can be obtained.

The coil self-capacitance *C* can be calculated through the electrostatic energy $E_{e}=\left(1/2\right)CU_{0}^{2}$, $U_{0}=R\;I_{0}$. Analytical expressions exist to evaluate the self-capacitance of a coil [39]. As the gap *s* between conductors is small, a simple expression of the capacitance can be written for a scroll coil by assuming that the capacitance consists of an association in series of N−1 capacitances, where *N* is the number of turns.

(5) $$\frac{1}{C}=\sum_{i=1}^{N-1}\frac{1}{C_{i}}$$

Each capacitance $C_{i}$ is formed by the *i*th turn with the (i+1)th turn, so that:

(6) $$C_{i}=\epsilon_{0}\epsilon_{r}\frac{2\pi \;a_{i}\times h}{s},$$

where $\epsilon_{r}$ is the relative permittivity of the dielectric filling the gap *s* and $a_{i}$ is the radius of the *i*th turn. As the $a_{i}$ vary slowly with *i*, an approximate expression of *C* can be obtained by replacing each $a_{i}$ by the average coil radius $a_{m}$. Thus:

(7) $$C\approx \epsilon_{0}\epsilon_{r}\frac{2\pi \;a_{m}\times h}{s}\frac{1}{N-1}$$

As the number of turns increases, the self-capacitance *C* tends to decrease, whereas *L* increases with *N*. Finally, additional turns slowly decrease the self-resonance frequency. For Coil 2, L≈1nH, C≈0.5 pF (calculated with $\epsilon_{r}=3$), giving a self-resonance frequency of ≈7 GHz, which is far above 200 MHz.

The insulator filling the gap between the conductors is the site of losses which are represented, by the parallel resistance $R_{p}$ in Figure 2a:

(8) $$R_{p}=\frac{1}{2\pi \;f\;C\;\tan\delta }$$

If the dissipation factor of the insulator is tanδ=0.01 for Coil 2, then $R_{p}\approx 160k\Omega$ at 200 MHz, which means that the dielectric losses are totally negligible compared to conductor losses.

#### 2.2.5. Comparison of 3D- and 2D-Axisymmetric Models

Table 1 shows the properties of Coil 1 and Coil 2, which are calculated with 2D-axisymmetric FEM and 3D-FEM. 2D-axisymmetric models systematically underestimate coil resistance by about 4% compared to 3D-models. On the contrary, the 2D-axisymmetric models overestimate the magnetic field of about 1.5%. The $B_{1}$-homogeneities of 2D- and 3D-models are in good agreement.

In conclusion, the 2D-modelling tends to overestimate SNR of about 4%. This estimation of the error was performed for 4-turns and some values of *w* and *s*. It is reasonable to think that the relative error will be smaller for a larger number of turns and smaller values of *w* and *s*. In any case, the SNR overestimate of 2D-FEM should be uniform over a narrow range of variations of the parameters, so that the retrieved optimum point should be weakly affected.

## 3. Coil Optimisation

### 3.1. Method

The problem to be solved is to find the coil parameters which maximize SNR with the contraint of $B_{1}$-homogeneity H>90%. The intensive use of FEM in the optimisation process would be very time consuming. Indeed, the accuracy of each model must be controlled to a high level at each step. Thus, an analytical model is preferrably used in an optimisation routine [40,41]. In order to make a parsimonious use of FEM, we combine FEM with the design of experiment (DOE) approach. This method permits to determine an interpolation function of the three coil parameters: *w*, *h* and *s*, for any response of interest (SNR, *R*, …). The interpolation function must have a simple form to be readily incorporated in an optimisation routine. We used a polynomial function derived from a Taylor expansion up to the third order around the point $P_{0}$ of coordinates ($w_{0}$, $h_{0}$, $s_{0}$). Such an interpolation function is valid only in the vicinity of $P_{0}$, so that several interpolation functions are necessary to cover a large domain. The third order Taylor expansion comprises 20 terms, but the six interactions of quadratic terms with linear terms ($w^{2}\;h$, $w^{2}\;s$, …) proved to be not significant, so that they were discarded.

**Figure 6:**
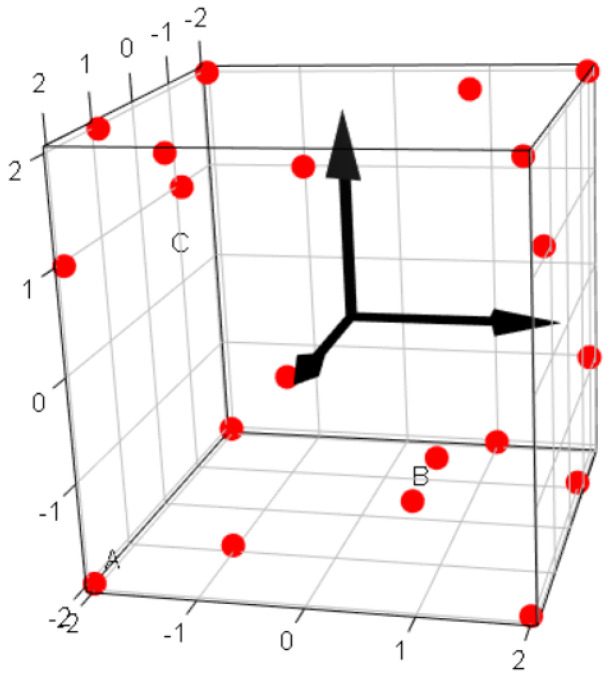
Cubic box representing the domain of variations of the parameters: *w*, *h* and *s*. The factors A, B and C represent centred non-dimensional values of parameters. The boundaries of the cube are at levels ±2. The 20 points define the 20 models of the design of experiment (DOE), from which the responses of interest (SNR, R, $<B_{z}>$, etc) are calculated.

Linear regression is used to determine the 14 coefficients of the interpolation function. To have the 14 coefficients with the minimum error, we calculate within a cubic domain the responses of interest at 20 particular points. Thus, 6 degrees of freedom are associated to residuals. An algorithm based on the theory of optimal experimental design of Fedorov [42] is used to find the 20 optimal points, represented in Figure 6. The algorithm is implemented in the function optFederov() included in the R package “AlgDesign” [43,44]. The algorithm selects 20 points among a list of $125=5^{3}$ candidate points in the cubic box, corresponding to the 5 levels: −2, −1, 0, 1, 2 tested on each axis. The design of experiment (DOE) comprises 20 points which can be readily executed through a parametric study in COMSOL Multiphysics^®^. The responses of interest are exported to calculate the 14 coefficients of the interpolation function by linear regression. Residuals are inspected to verify the prediction accuracy of the interpolation function. It is then incorporated in the optimisation routine to find the point ($w_{opt}$, $h_{opt}$, $s_{opt}$) which maximise SNR. If the optimum point is found at a boundary of the cubic box, it means that the optimum has not yet been found. A new interpolation function is calculated which is valid in the new domain next to the preceding cube. If the optimal solution is found within the current domain, then the program is stopped. With this step-by-step method, the evolution of the optimisation process is traced from the beginning to the end. More details about the method presented here can be found in the Appendix A.

### 3.2. Result of Optimisation at 200 MHz for a Nanoliter Sample

The number of turns being fixed initially, the method of optimisation described in the preceding section was applied. The optimal coil parameters were calculated for a sample of 1 nL and a coil working at 200 MHz. The results are presented in Table 2 for the number of turns varying from 2 to 15. The optimum trace widths $w_{opt}$ are in the range of 2–7 μm, which are of the order of the skin-depth δ at 200 MHz. The optimum trace height $h_{opt}$ are in the range of 80–115 μm, which matches the sample volume. Surprisingly, we found no optimum for the parameter *s*, so the results of Table 2 are presented for the arbitrary value s=1 μm. In fact, SNR linearly increases as *s* tends to zero in the vicinity of the point ($w_{opt}$, $h_{opt}$). The decrease of *s* from 1 μm to 0.5 μm increases SNR marginally of about 1% in the worst cases. No significant variation of SNR with *s* exists for coils of less than 5 turns. It turns out that scroll microcoils do not suffer proximity effect. This feature permits to choose the smallest possible value of *s* to have the maximum SNR. However, a very low value of *s* would marginally enhance SNR, but would not ensure a good electrical insulation between the conductors. Also, the coil self-capacitance, which increases as 1/s as *s* tends to zero, would be considerably increased for a very small value of *s*. The consequence of a very low value of *s* is the increase of the capacitance, the lowering of the self-resonance frequency and an increase of dielectric losses through the lowering of the parallel resistance $R_{p}$ in Equation (8).

Table 2 shows that SNR and $B_{1}$-homogeneity increase monotonically with the number of turns from 2 to 10, but begins to decrease after 10 turns. The 12-turns scroll coil has a resistance of about 1Ω, which is sufficient to preserve the intrinsic SNR of the coil when it is connected to the readout circuit [16]. The self-capacitance and the self-inductance of the 12-turns coil with s=1 μm are 0.15pF ($\epsilon_{r}=3.3$) and 8.4nH, giving the self-resonance frequency 4.5 GHz. The self-resonance frequency is high enough to use the coil for broadband multinuclei detection [16].

**Figure 7:**
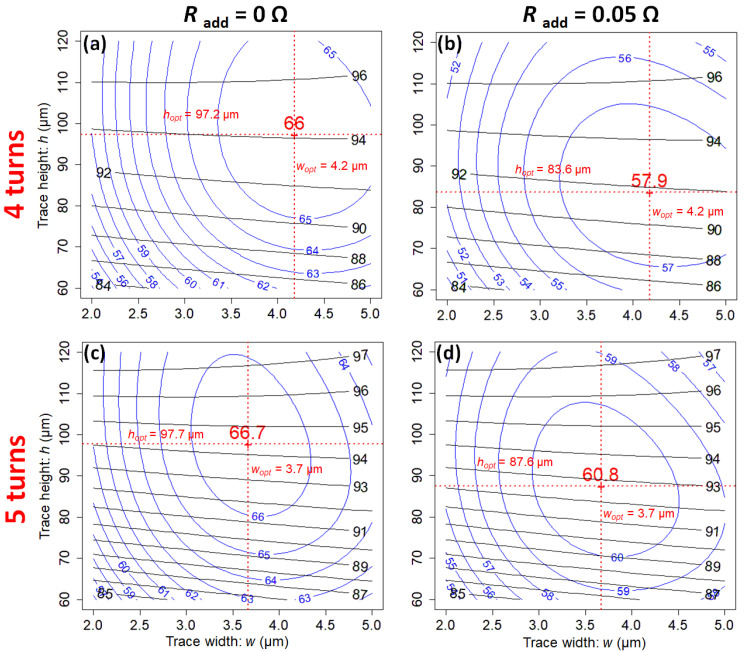
Contour plots of SNR (in $mT\;A^{-1}\;\Omega^{-1/2}$ ) at 200 MHz (blue solid lines), versus trace width *w* and trace height *h*, for the gap s=1 μm, for a scroll coil of 4-turns (**a**,**b**) and 5-turns (**c**,**d**). The optimum coil parameters depend on the additional resistance $R_{add}$. Subplots (**a**,**c**) are for the intrinsic coil resistance and subplots (**b**,**d**) are for $R_{add}=50\;m\Omega$. Contour plots for $B_{1}$-homogeneity are superimposed (black solid lines).

Figure 7 shows contour plots of SNR and *H* as a function of *w* and *h*, for s=1 μm around the optimum solutions, for 4 and 5 turns. Figure 7b,d show the effect of an additional resistance of 50mΩ, due to connexion wires, possible sample losses or dielectric losses. Figure 7a,c concern the intrinsic coil resistance. SNR is less affected by this additional resistance when the resistance has the greatest value.

The optimum trace width $w_{opt}$ does not depend on the additional resistance, contrary to the optimum trace height $h_{opt}$ which tends to decrease. To minimize the effect of additional resistance, the intrinsic coil resistance *R* should be high. In the perspective of using scroll microcoils for broadband multinuclei NMR, where the low noise amplifier can be directly connected to the coil without impedance matching and without tuning, it is mandatory to have the highest coil resistance in order to preserve the intrinsic SNR of the coil [16,20].

Beyond 10 turns, the SNR begins to decrease. As $\mathrm{SNR}\propto B_{1}/\sqrt{R}$, the decrease in SNR means that the denominator $\sqrt{R}$ increases faster than the numerator $B_{1}$. Indeed, the contribution to the $B_{1}$-field of outer windings is becoming smaller and smaller as the windings radii increase, whereas the resistance of these windings increases. From 10 to 15 turns, the increase of $\sqrt{R}$ is about 44%, which is almost compensated by the increase of 43% of $B_{1}$. This results in a decrease in SNR of only 1% from 10 to 15 turns. To decide which coil is optimum, other criteria than SNR must be applied. In order to preserve the intrinsic SNR, it may be judicious to favour a large coil resistance and thus to favour a coil with the largest number of turns. In addition, a large number of turns is beneficial to $B_{1}$-homogeneity. However, the addition of turns increases the inductance of the coil, which lowers the self-resonance frequency and risks compromising the use of the coil for broadband NMR spectroscopy. Anyway, depending on the intended application, the optimum number of turns would be between 10 and 20. A large number of turns gives the coil a good mechanical resistance which avoids the use of a support, so that the coil could serve as a container for the sample.

## 4. Discussion of Results

### 4.1. Coil Resistance

**Figure 8:**
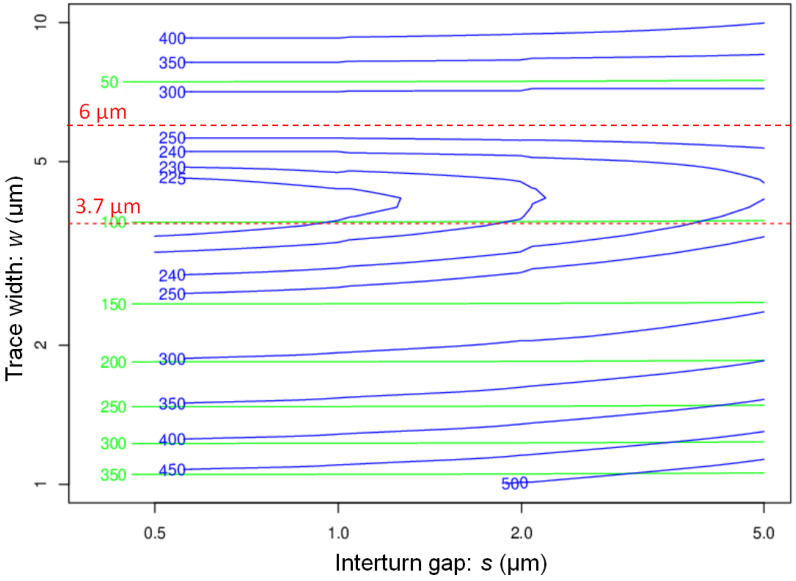
Contour plots of intrinsic DC and AC resistance at 200 MHz in mΩ, for a scroll coil of 5 turns, versus gap *s* and trace width *w*, for a trace height h=100 μm. The almost horizontal green lines are the DC resistance.

Figure 8 shows the contour plot of DC and AC resistance for a coil of 5-turns as a function of *w* and *s*, for h=100 μm, at 200 MHz. The lines of iso-DC resistance are almost horizontal, which shows that the DC resistance is weakly *s*-dependant, contrary to the AC resistance. The contour plot of AC resistance displays a “valley of low resistance” at w≈4 μm which gently slopes down towards s≈0. The minimum of resistance is about three times the DC resistance for w=4 μm, which is of the order of the skin-depth δ at 200 MHz. The variation of AC resistance of scroll coils is thus very different from that of flat planar coils, where the AC resistance may be 100 times larger than the DC resistance [45]. For a trace width $w>w_{min}$, the AC resistance grows rapidly with *w*. For w≈10 μm, the AC resistance is about 10 times the DC resistance.

In the usual proximity effect, the AC resistance decreases when the distance between conductors increases. This behaviour is only observed for w>2δ≈6 μm. When *w* grows from 4 to 8 μm, the current distribution becomes very inhomogeneous (compare Figure 3 and Figure 4), which increases the AC resistance.

In the valley and on the side w<4 μm, the proximity effect is unusual; the AC resistance grows as *s* increases. For w=4 μm, there is about 10% increase of AC resistance when the gap *s* varies from 0.5 to 5 μm. This unusual proximity effect was already pointed out by Grant et al. [23]. The unusual proximity effect suffered by scroll coils is beneficial to SNR, as it allows the fabrication of a compact coil with a relatively low AC resistance and a high $B_{1}$ field.

### 4.2. Comparison with a Solenoid

**Figure 9:**
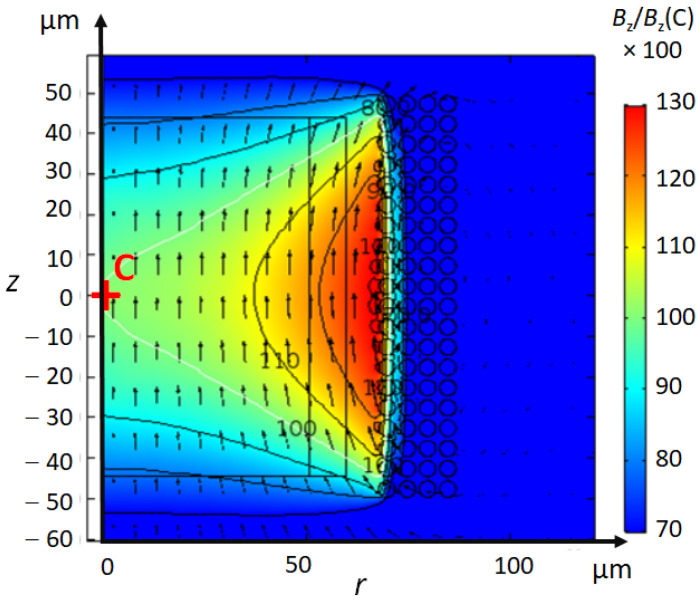
2D-axisymmetric Finite Element Modelling (FEM) of the solenoid. The vertical axis r=0 is the axis of symmetry of the coil. Magnetic field inside a solenoidal coil built with 80 turns if a copper wire of diameter 4 μm distributed uniformly over 4 layers of 20 turns each with a gap of 1 μm between adjacent wires. The height of the solenoid is thus 100 μm. The contour plot shows the relative magnitude of the real part of the $B_{z}$-component in percent. The contour line in white represents the line 100%, the value of $B_{z}=538.7\;mTA^{-1}$ at the centre of the coil. In the sample region of volume 1nL, the magnetic field of the solenoid is a bit less homogeneous than the scroll coil of Figure 5.

The homogeneity of the magnetic field is similar for scroll coils and solenoids. It is instructive to compare the SNR of both coils for the same sample volume and the same working frequency. For the comparison, we chose as reference, a scroll coil of 4-turns, named Coil 3, which is identical to Coil 2, excepted that the gap *s* is 1 μm instead of 2 μm. Coil 3 is close to the optimum scroll coil with 4 turns in Table 2. We compare Coil 3 to a solenoid of the same dimensions, i.e., with 4-layers of 20 turns of wires of diameter 4 μm, separated by a gap of 1 μm, that is shown in Figure 9. The length of the solenoid is then 100 μm. In fact, the fabrication of such a microsolenoid with such a thin wire would be very difficult. Figure 9 shows the magnetic field inside the solenoid. The comparison of performance for the solenoid and Coil 3 is presented on Table 3. $B_{1}$-homogeneity is slightly better for the scroll coil. The current distribution of the solenoid is determined by the distribution of wires, as the current density is almost uniform within the wires. The reinforcement of current density at the top and bottom of a scroll coil favours $B_{1}$-homogeneity.

The main properties of coil performance: SNR, $B_{1}$-homogeneity and quality factor of both coils are of the same order of magnitude, although the scroll coil has a much lower resistance.

### 4.3. Coil Optimisation for a Subnanoliter Sample

Let us consider the 12-turns scroll microcoil optimised for a sample of 1 nL at 200 MHz. This coil has an inner turn radius $a_{1}=70$ μm, a trace width of 2.2 μm and a trace height of 111 μm. If the sample volume is changed, the coil dimensions must be changed to match the sample volume and the optimum coil parameters have to be calculated again. Nevertheless, it is possible to use the scaling law which binds coil dimensions and the working frequency *f*. If the coil dimensions are multiplied by a factor of α, then the resulting current distribution within the conductor will be similar, provided that the working frequency is divided by $\alpha^{2}$. This assertion is equivalent to say that the skin-depth $\delta \propto 1/\sqrt{f}$ governs the current distribution. The similarity of the current distribution means that the new similar coil will be optimum at the new frequency. For example, let us suppose that α=1/2, then the new similar coil, with $a_{1}=35$ μm, will be optimised for a sample of 1/8nL and a working frequency of 800 MHz. On the other hand, if α=2, then the new scroll coil will have $a_{1}=140$ μm and optimised for a sample volume of 8nL and the new working frequency of 50 MHz.

As $\mathrm{SNR}\propto f^{2}\;V_{s}\sqrt{G}\;<B_{z}>/I$ (Equation (1)), the rescaling of coil properties with the coil dimension $a_{1}$ can be derived as follows: frequency $f\propto a_{1}^{-2}$, sample volume $V_{s}\propto a_{1}^{3}$, coil conductance $G\propto a_{1}$ (resistance $R\propto 1/a_{1}$) and magnetic field $<B_{1}>\propto 1/a_{1}$. Thus,

(9) $$\mathrm{SNR}\propto a_{1}^{-3/2}\propto f^{3/4},$$

so the miniaturisation of scroll coils is beneficial to SNR. In addition, self-inductance $L\propto a_{1}$, quality factor Q∝1, self-capacitance $C\propto a_{1}$ and self-resonance frequency $f_{r}\propto 1/a_{1}$. Coil miniaturisation has no effect on quality factor but it increases the self-resonance frequency. Also, the parallel resistance $R_{p}\propto a_{1}$, which means that miniaturisation increases dielectric losses relatively to conductor losses. It is possible that a low loss dielectric such as Teflon would be necessary at very high frequencies.

## 5. Conclusions and Perspectives

We presented a method of optimisation of the design of scroll microcoils. These coils could be built by rolling up a thin bilayer of copper/insulator foil. We performed optimisation for a sample of 1 nL at 200 MHz. Though the result of optimisation is dependant on both sample volume and working frequency, a scaling law can simplify the search of the optimum coil for other sample volumes. The optimal scroll microcoil has a number of turns greater than 10, which should give the coil a good mechanical strength and thus avoid the use of a rigid support to hold the coil. Moreover, the large number of turns is beneficial to $B_{1}$-homogeneity and the resulting high electrical resistance (>1 Ω) facilitates the adaptation of the coil to the low-noise amplifier, in the perspective of using the coil for non-tuned broadband NMR. The high self-resonance frequency is favourable to broadband spectroscopy. The performance of scroll coils and solenoids in terms of SNR and $B_{1}$-homogeneity are similar, but the batch microfabrication of scroll microcoils designed for subnanoliter sample could be easier.

## Figures and Tables

**Table 1:** Results of 2D-axisymmetric FEM and 3D-FEM of Coil 1 and Coil 2 at 200 MHz (see Figure 3 and Figure 4 for coil parameters). The relative difference of signal-to-noise ratio (SNR) between the two models is less than 4% for both coils.

	Model	*R* (mΩ)	$B_{z}$ (C) (mT A${}^{-1}$)	$<B_{z}>$ (mT A${}^{-1}$)	H(%)	SNR (mT A${}^{-1}$Ω${}^{-1/2}$)
	2D	201.2	23.61 + 0.23 i	24.44 + 0.26 i	94.9	54.5
Coil 1	3D	210.9	23.68 + 0.24 i	24.39 + 0.27 i	94.4	53.1
	relative difference	−4.6%	−0.3%	0.2%	0.5%	2.6%
	2D	156.67	24.80 − 0.49 i	25.56 − 0.52 i	94.8	64.6
Coil 2	3D	163.51	24.44 − 0.46 i	25.20 − 0.50 i	94.1	62.3
	relative difference	−4.2%	1.5%	1.4%	0.7%	3.6%

**Table 2:** Coil parameters for optimum SNR at 200 MHz for a cylindrical sample of volume $V_{s}=1nL$ of radius $r_{s}=60$ μm and height $h_{s}=88.4$ μm. The inner turn radius $a_{1}=70$ μm and the gap s=1 μm are fixed parameters.

Number	Optimum Trace	Optimum Trace	Coil	Average MF	$B_{1}$-homogeneity	SNR	Self-
of Turns	Width	Height	Resistance	Within Sample	Equation (2)	$<B_{z}>/\sqrt{R}$	Inductance
$N_{t}$	w(μm)	h(μm)	*R* (Ω)	$<B_{z}>$ (mT A${}^{-1}$)	H(%)	(mT A${}^{-1}$ Ω ${}^{-1/2}$)	*L* (nH)
2	6.74	92.9	0.0482	13.948 − 0.154 i	92.9	63.6	0.223
3	4.99	98.0	0.0941	19.927 − 0.322 i	94.0	65.0	0.502
4	4.20	97.2	0.1564	26.151 − 0.457 i	94.2	66.1	0.915
5	3.67	97.7	0.2300	32.082 − 0.592 i	94.5	66.9	1.449
6	3.29	99.5	0.3126	37.694 − 0.742 i	94.9	67.4	2.091
7	3.00	101.7	0.4038	43.044 − 0.893 i	95.3	67.7	2.846
8	2.77	104.2	0.5023	48.133 − 1.040 i	95.8	67.9	3.865
10	2.45	106.0	0.7397	58.478 − 1.303 i	96.3	68.0	5.863
12	2.19	111.5	0.9894	67.429 − 1.583 i	97.1	67.8	8.366
15	1.99	107.5	1.5365	83.426 − 1.899 i	96.8	67.3	13.73

**Table 3:** Comparison of performance at 200 MHz of the solenoid microcoil of Figure 9 and a scroll Coil 2 with parameters: 4-turns, $a_{1}=70$ μm, w=4 μm, h=100 μm, and s=1 μm. For both coils, the sample dimensions (volume 1nL) are identical.

Coil Type	*R* (mΩ)	$B_{z}(C)$ (mT A${}^{-1}$)	$<B_{z}>$ (mT A${}^{-1}$)	*L* (nH)	$Q=\frac{L\omega }{R}$	H(%)	SNR (mT A${}^{-1}$ Ω ${}^{-1/2}$)
4-layers solenoid	57.845	538.7 + 0.35 i	557.6 + 0.40 i	383.4	8.33	89.6	73.3
4-turns scroll Coil 3	0.1536	25.19 − 0.48 i	25.91 − 0.50 i	0.900	7.37	94.6	66.1

**Figure A1:**
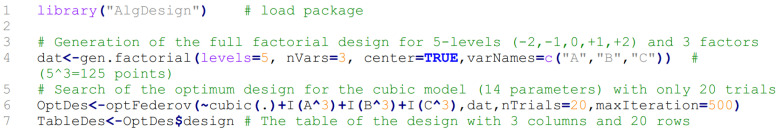
Script using the functions gen.factorial() and optFederov() to find the optimum design for the interpolation function of 14 terms. A design of 20 trials permits to have 6 degrees of freedom for the residuals. The analysis of residuals is mandatory to test the validity of the model which will be used for optimisation.

**Figure A2:**
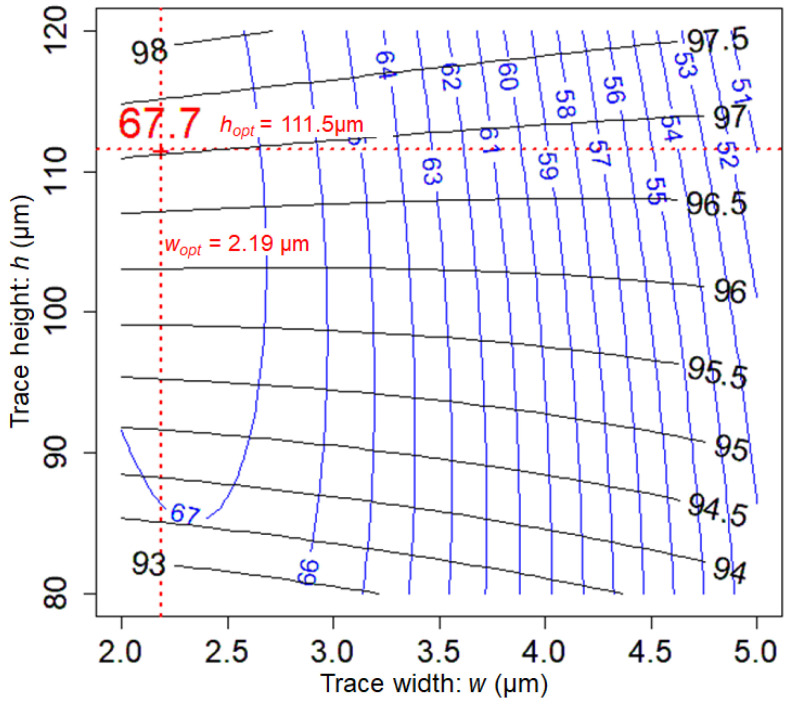
Contour plot of SNR, in $mT\;A^{-1}\Omega^{-1/2}$ (blue lines), as a function of trace width *w* and trace height *h*, for a 12-turns scroll coil, at 200 MHz and for s=1 μm. The contour plot of the response *H* ($B_{1}$-homogeneity), in percent, is superimposed (black dotted lines).

## Data Availability

The data presented in this study are available on request from the corresponding author.

## Abbreviations

The following abbreviations are used in this manuscript:

DOE	Design of Experiment
FEM	Finite Element Modelling
LOD	Limit of detection
MRI	Magnetic resonance imaging
NMR	Nuclear Magnetic Resonance
SNR	Signal-to-noise ratio

## Appendix A. The R-Package “AlgDesign”

In order to minimize the number of models built with COMSOL Multiphysics^®^, we used an optimum experimental design to determine the coefficients of the following interpolation function *Y* (model of the response) of the 3 factors A, B and C:

$$\begin{array}{ccc} Y= & a_{1} & \;(\mathrm{constant})\\ \; & +\;a_{2}\;A+a_{3}\;B+a_{4}\;C & \;(\mathrm{linear}\;\mathrm{terms})\\ \; & +\;a_{5}\;A^{2}+a_{6}\;B^{2}+a_{7}\;C^{2} & \;(\mathrm{quadratic}\;\mathrm{terms})\\ \; & +\;a_{8}\;A^{3}+a_{9}\;B^{3}+a_{10}\;C^{3} & \;(\mathrm{cubic}\;\mathrm{terms})\\ \; & +\;a_{11}\;\mathrm{AB}+a_{12}\;\mathrm{AC}+a_{13}\;\mathrm{BC} & \;(2-\mathrm{factors}\;\mathrm{interaction}\;\mathrm{terms})\\ \; & +\;a_{14}\;\mathrm{ABC} & \;(3-\mathrm{factors}\;\mathrm{interaction}\;\mathrm{term}). \end{array}$$

The response *Y* may be: SNR, $B_{1}$-homogeneity, or any response of interest. The factors A, B and C are the coil parameters: *w*, *h* and *s*. The optimum design was found by using the package “AlgDesign” of R [43]. Figure A1 shows the R-program used to calculate the optimum DOE.

Table A1 shows the optimum DOE.

**Table A1:** Optimum DOE for 3 factors comprising 20 trials, that are suitable to find the 14 coefficients $a_{i}$ of the model (interpolation function). Coil parameters *w*, *h* and *s* are given as coded values. The coded values can be readily converted into real values by a linear transformation once the levels −1 and +1 are specified for each parameter.

Trial Number	1	2	3	4	5	6	7	8	9	10	11	12	13	14	15	16	17	18	19	20
Trace width: *w*: A	−2	2	1	−2	−1	2	−1	1	2	−2	−1	2	−2	1	−2	1	2	−1	−2	2
Trace height: *h*: B	−2	−2	−1	1	2	2	−1	1	1	2	−2	−2	−1	2	−2	−2	−1	1	2	2
Spacing: *s*: C	−2	−2	−2	−2	−2	−2	−1	−1	−1	−1	1	1	1	1	2	2	2	2	2	2

The design is integrated as a parametric study in the FEM software. The responses of interest of the 20 FEM simulations are shown for a 12-turns scroll coil in Table A2. Using the column SNR of Table A2, the coefficients of the interpolation function are calculated using linear regression. The significant coefficients are shown in Table A3. Factor C (spacing *s*) is only present in the linear effect (−0.831) and the coupling AC (0.206). As the level of A is about −2 for the optimum coil ($w_{opt}\approx 2$ μm), the effect of factor C is about −1.25 at the optimum, which means that SNR varies of −1.25 as the level of *s* varies from 0 to 1 (in real values *s* varies from 1.5 to 1.75 μm). As the effect of *s* is linear (no quadratic or cubic terms), SNR decreases of about $5\;mT\;A^{-1}\Omega^{-1/2}$ as *s* varies from 1 to 2 μm, i.e., about 10%. In consequence, the reduction of the spacing between the conductors is beneficial to SNR. The increase of SNR as *s* decreases is mainly due to the favourable proximity effect discussed in Section 4.1.

**Table A2:** Output properties, at 200 MHz, of the 20 scroll microcoils with 12-turns, according to the DOE of Table A1. The inner turn radius $a_{1}=70$ μm is identical for all coils. The properties are calculated by using 2D-axisymmetric FEM.

Trial	*w*	*h*	*s*	*R*	$<B_{z}>$	$B_{z}$ (C)	H	SNR	*L*	*C*	$f_{r}$
Number	μm	μm	μm	Ω	mT A${}^{-1}$	mT A${}^{-1}$	%	mT A${}^{-1}$ Ω ${}^{-1/2}$	nH	pF	GHz
1	2	80	1	1.3590	77.168 − 1.321i	74.204 − 1.300i	92.5	66.2	9.84	0.115	4.72
2	5	80	1	1.6103	67.630 + 0.474i	66.638 + 0.260i	93.8	53.3	10.8	0.137	4.14
3	4.25	90	1	1.3282	67.187 − 0.217i	65.099 − 0.231i	94.8	58.3	10.0	0.148	4.13
4	2	110	1	1.0266	68.497 − 1.671i	66.661 − 1.438i	96.9	67.6	8.37	0.159	4.37
5	2.75	120	1	0.9110	63.438 − 1.297i	62.147 − 1.109i	98.0	66.5	8.22	0.181	4.12
6	5	120	1	1.3596	58.356 + 0.156i	57.191 + 0.223i	97.5	50.0	8.99	0.206	3.70
7	2.75	90	1.25	1.1669	70.879 − 1.152i	68.530 − 1.085i	94.6	65.6	9.61	0.110	4.89
8	4.25	110	1.25	1.1943	61.670 − 0.397i	60.283 − 0.306i	96.8	56.4	9.22	0.147	4.32
9	5	110	1.25	1.4124	60.127 + 0.173i	58.819 + 0.214i	96.8	50.6	9.48	0.153	4.18
10	2	120	1.25	0.9656	64.999 − 1.686i	63.488 − 1.438i	98.4	66.1	8.07	0.141	4.72
11	2.75	80	1.75	1.3076	71.933 − 1.026i	69.187 − 1.004i	93.3	62.9	10.3	0.072	5.83
12	5	80	1.75	1.6061	65.792 + 0.384i	64.868 + 0.178i	94.0	51.9	11.1	0.082	5.29
13	2	90	1.75	1.2929	71.932 − 1.523i	69.494 − 1.438i	94.6	63.3	9.57	0.078	5.84
14	4.25	120	1.75	1.1456	58.326 − 0.458i	57.145 − 0.335i	97.6	54.5	9.04	0.118	4.88
15	2	80	2	1.4482	73.750 − 1.358i	71.154 − 1.368i	93.0	61.3	10.2	0.061	6.36
16	4.25	80	2	1.4290	66.961 − 0.187i	64.842 − 0.254i	93.8	56.0	10.9	0.070	5.77
17	5	90	2	1.5334	63.091 + 0.264i	61.273 + 0.207i	95.2	50.9	10.7	0.081	5.40
18	2.75	110	2	1.0335	63.614 − 1.340i	62.105 − 1.162i	97.3	62.6	9,00	0.088	5.65
19	2	120	2	1.0195	63.141 − 1.727i	61.824 − 1.497i	99.1	62.5	8.36	0.092	5.74
20	5	120	2	1.3604	56.513 + 0.057i	55.464 + 0.143i	97.6	48.5	9.41	0.109	4.98

**Table A3:** Coefficients and standard deviation of coefficients $a_{i}$, in $mT\;A^{-1}\Omega^{-1/2}$, of the interpolation function for the response SNR, which was calculated by linear regression from the data of Table A2. The maximum of the residuals is about $0.2\;mT\;A^{-1}\Omega^{-1/2}$, which is sufficiently small to expect accurate evaluation of SNR.

	Constant	A	B	C	A${}^{2}$	B${}^{2}$	C${}^{2}$	A${}^{3}$	B${}^{3}$	C${}^{3}$	AB	AC	BC	ABC
$a_{i}$	61.4	−4.25	−0.237	−0.831	−0.873	−0.113	–	0.211	–	–	−0.279	0.206	–	–
Standard deviation	0.081	0.057	0.016	0.016	0.019	0.019	–	0.015	–	–	0.009	0.009	–	–

Using the coefficients of the interpolation function for the response SNR, the contour plot of SNR versus *w* and *h* is drawn for s=1 μm (Figure A2). The optimum coil parameters that are shown in Figure A2 are those of Table 2. The contour plot for the response *H* ($B_{1}$-homogeneity) is superimposed in Figure A2.
